# Role of GABA_B_ receptors and p38MAPK/NF-κB pathway in paclitaxel-induced apoptosis of hippocampal neurons

**DOI:** 10.1080/13880209.2017.1392987

**Published:** 2017-11-08

**Authors:** Zhao Li, Peng Liu, Hailin Zhang, Shuang Zhao, Zi Jin, Rui Li, Yuexian Guo, Xiuli Wang

**Affiliations:** a Department of Anesthesiology, The Third Hospital of HeBei Medical University, Shijiazhuang, HeBei Province, China;; b Department of Pharmacology, HeBei Medical University, Shijiazhuang, HeBei Province, China

**Keywords:** Inhibition rate, neuropathic pain model, Morris water maze, western blot, immunohistochemistry assay

## Abstract

**Context:** The effects of the anticancer drug paclitaxel on learning and memory are rarely studied.

**Objective:** This study investigated changes in GABA_B_ receptor expression during paclitaxel-induced apoptosis of hippocampal neurons and the role of the p38MAPK/NF-κB pathway in this process.

**Materials and methods:** Hippocampal neurons isolated from neonatal Sprague–Dawley rats were divided into six groups: Control (C), SB (10 µL of 10-µmol/L SB203580), SN (53 µg/mL SN50), N (1 µmol/L paclitaxel), SB + N (10 µmol/L SB203580 + 1 µmol/L paclitaxel) and SN + N (53 µg/mL SN50 + 1 µmol/L paclitaxel). Cells in different groups were treated with corresponding agents for 24 h at 37 °C. The apoptosis rate and protein levels of GABA_B1_ receptors and NF-κB p65 were evaluated. Rat models of neuropathic pain was induced by paclitaxel and were divided into four groups such as N, B + N, SN + N and SN + B + N groups. Rats in the N group received intrathecal injections of normal saline solution. Rats in the B + N group received intrathecal injections of 10 μL baclofen (0.05 μg/μL). Rats in the SN + N and SN + B + N groups received intrathecal injections of SN50 and SN50 plus baclofen, respectively. Spatial learning and memory were evaluated in rat models based on the escape latency and the number of crossings over the platform and protein levels of GABA_B1_ receptors, NF-κB, IL-1β and TNFα were measured by immunohistochemistry assay and western blot.

**Results:** The neuronal apoptosis rate was significantly increased in N (49.16 ± 3.12)%, SB + N (31.18 ± 3.02)% and SN + N (28.47 ± 3.75)% groups, accompanied by increased levels of GABA_B1_ receptors and NF-κB p65 (*p* < 0.05). The paclitaxel-treated rats demonstrated significantly increased latency (24.32 ± 2.94)s and decreased the crossings number (3.14 ± 0.63) after 15 d in the Morris water maze (*p* < 0.05). Immunohistochemistry assay showed that compared with the N group (GABA_B1:_9.0 ± 1.6, NF-κB p65:29.6 ± 2.4, IL-1β: 30.4 ± 3.4, TNFα: 31.0 ± 3.4), B + N, SN + N and SN + B + N groups evidently increased levels of GABA_B1_ receptor (B + N:SN + N:SN + B + N = 19.4 ± 2.1:20.8 ± 1.9:28.0 ± 1.9) but significantly decreased levels of NF-κB p65 (B + N:SN + N:SN + B + N = 21.2 ± 1.5:18.6 ± 2.1:12.6 ± 1.5), IL-1β (B + N:SN + N:SN + B + N = 22.0 ± 1.0:19.6 ± 1.8:14.6 ± 1.5) and TNF α (B + N:SN + N:SN + B + N = 23.0 ± 1.6:22.2 ± 0.8:16.6 ± 1.7). Similar findings were found in western blot analysis.

**Discussions and conclusions:** Paclitaxel may reduce cognitive function in rats through the p38MAPK/NF-κB pathway and GABA_B1_ receptors.

## Introduction

Paclitaxel, a well-known anticancer drug, causes sensory and peripheral motor neurotoxicity in patients (Frederiks et al. 2015). However, the effects of paclitaxel on learning and memory remain unclear. The hippocampus, cortex and striatum are core regions in the limbic system (Rolls 2015). The hippocampus is located under the cerebral cortex and plays a critical role in spatial memory, navigation, learning and other cognitive processes (Czerniawski et al. 2014; Kanoski and Grill 2015).

Neuronal apoptosis and neuropathies induced by paclitaxel may involve the mitogen-activated protein kinase (MAPK) pathway, which is involved in cancers (McDaid and Horwitz 2001; Taxman et al. 2003). The p38MAPK subfamily of the MAPK family triggers inflammatory responses and endothelial cell apoptosis. Previous studies have suggested that the p38MAPK/NF-κB pathway mediates T cell immune responses (Liu et al. 2014) and apoptosis (Chiu et al. 2010). Further, NF-κB expression and activity are increased in neurons surviving hippocampal injury (Kassed et al. 2004) and are required for memory formation in the hippocampus (Jarome et al. 2014). GABA_B_ receptors, which are G-protein-coupled receptors, are crucial in ameliorating cognitive and memory impairment (Li et al. 2014, 2016) in neuropsychological disorders associated with the hippocampus. GABA_B_ receptors regulate TLR4- and TLR3-induced nuclear expression of NF-κB p65 (Crowley et al. 2015). GABA also suppresses NF-κB activation in human immune cells (Prud'Homme et al. 2013). These studies have revealed the interaction between GABA_B_ receptors and the NF-κB pathway. Therefore, the NF-κB pathway and GABA_B_ receptors, as well as p38MAPK, are important in hippocampal neuronal apoptosis and neuropathy induced by paclitaxel.

In this study, the p38MAPK inhibitor SB203580, the NF-κB translocation inhibitor SN50 and the GABA_B_ receptor agonist baclofen were used to investigate the effects of paclitaxel on GABA_B_ receptor expression. In addition, the regulatory effects of the p38MAPK/NF-κB pathway in this process were explored. Our results may illuminate the mechanisms underlying paclitaxel-induced neuropathic pain and learning and memory impairment.

## Materials and methods

### Primary cultures of rat hippocampal neurons

Approval was obtained from the Animal Care and Use Committee of Hebei Medical University, and all animal experimental protocols were performed in accordance with ethical standards. Newborn Sprague–Dawley (SD) male rats <24 h old were purchased from the Experimental Animal Research Center of Hebei Medical University (Hebei, China). All rats were sacrificed within 24 h after birth and the skin was processed with 75% ethanol. After dissecting the skin and skull, hippocampi were separated from the brain. Neurons were separated from hippocampal tissues using papain (Acros, Basel, Switzerland), digestion and mechanical isolation. Dissociated hippocampal neurons were cultured in 96-well plates in Neurobasal medium (Invitrogen, Carlsbad, CA) supplemented with penicillin–streptomycin (Gibco, Carlsbad, CA) and B27 (Invitrogen, Carlsbad, CA) at 37 °C under 5% CO_2_ for five consecutive days. Half of the medium was freshly substituted every 48 h.

### Determining the optimal concentration of paclitaxel by MTT assay

After neurons were cultured for 5 d, paclitaxel (Sigma, St. Louis, MO) was added to the medium at final concentrations of 0, 0.01, 0.1, 1 and 10 µmol/L. MTT (20 μL) was added to each well 12, 24, 48 and 96 h after treatment. Each experiment was performed in quadruplicate. After incubating cells for 24 h, 150 µL DMSO was added to each well and the optical density was measured at 490 nm. The inhibition rate of cell proliferation was calculated as follows: inhibition rate (%) = (1 − experimental absorbance value/control absorbance value) × 100%. The half maximal inhibitory concentration (IC_50_) value was calculated using the improved Karber’s method: lgIC_50_ = *Xm* − I [*P* − (3 − *Pm* − Pn)/4], where *Xm* is the lg maximum dose, *I* is the lg maximum dose/adjacent dose, *P* is the sum of the positive reaction rates, *Pm* is the maximum positive reaction rate and *Pn* is the minimum positive reaction rate. This experiment was repeated three times.

### Apoptotic effects of paclitaxel detected by flow cytometry

After culturing for 5 d, hippocampal neurons were plated in six-well plates with paclitaxel at final concentrations of 0, 0.01, 0.1, 1 and 10 µmol/L. After 24 h of treatment, cells were digested by pancreatin and incubated with annexin V-FITC solution (Sigma, St. Louis, MO) for 15 min in the dark. Subsequently, cells were stained with propidium iodide (PI) and annexin V-FITC and analyzed on a FACScan flow cytometer (Sigma, St. Louis, MO). The excitation wavelength was 488 nm, and the fluorescence of FITC and PI was measured at 515 and 530 nm, respectively.

### Groups and treatment

After culturing for 5 d, hippocampal neurons (1 × 10^9^/L) were randomly divided into six groups: the no-treatment control (C) group, the SB group [10 µmol/L SB203580 (Sigma, Sigma, St. Louis, MO)], the SN group [53 µg/mL SN50 (Enzo, Lausen, Switzerland)], the N group (1 µmol/L paclitaxel), the SB + N group (10 µmol/L SB203580 + 1 µmol/L paclitaxel) and the SN + N group (53 µg/mL SN50 + 1 µmol/L paclitaxel). Cells in each group were treated for 24 h at 37 °C. After treatment, morphological changes in hippocampal neurons were observed using an inverted fluorescent microscope (Olympus America, Hauppauge, NY).

### Protein levels of GABA_B1_ receptors and NF-κB p65 determined by western blot assays

Hippocampal neurons from each of the six groups were lysed using cold RIPA lysis buffer containing PMSF and phosphatase inhibitor. Protein concentrations of GABA_B1_ receptors and NF-κB p65 were measured using the Lowry protein assay kit. Briefly, protein homogenates were resolved using SDS-PAGE and transferred onto PVDF membranes. Membranes were blocked in 5% skim milk powder for 1 h and incubated with primary antibodies (anti-GABA_B1_ receptor, 1:500; anti-NF-κB p65, 1:500; Santa Cruz, Santa Cruz, CA) at 4 °C overnight, followed by incubation with anti-rabbit IgG antibody conjugated to IRDye 800 (Rockland, Limerick, PA) for 4 h at room temperature. GAPDH was used as a loading control. Relative protein levels were determined using gray values detected by a two-color infrared imaging system (LI-COR; Lincoln, NE).

### Cell apoptosis measured by flow cytometry

Rat cells in the six groups were cultured in B27/N2 Neurobasal medium for 24 h. To determine the levels of hippocampal neuron apoptosis, a flow cytometry assay was performed as described above.

### Rat models of paclitaxel-induced neuropathic pain

Total 50 male SD rats weighing 160–180 g were adapted to the experimental environment at 20–25 °C, with free access to food and water for at least 1 week. The rat model of paclitaxel-induced neuropathy was constructed as previously described (Polomano et al. 2001). Briefly, neuropathic pain was induced in rats by four consecutive intraperitoneal injections of paclitaxel (2 mg/kg) every other day. The mechanical withdrawal threshold (MWT) was determined using a von Frey hair filament (Stoelting Wood, Wood Dale, IL) before treatment and at 9, 11 and 14 d (T1) after treatment. The 50% MWT was calculated as previously described (Dixon 1980). The animal model was successfully developed with an MWT of <6 g. Rats in the C group were treated with the same amount of normal saline solution.

### Morris water maze test (MWM)

The MWM test was performed to assess spatial learning and memory in experimental animals. This study included 15 rats with neuropathic pain (the P group) and 15 age-matched control rats (the C group). The maze was a round tank (150 cm diameter, 50 cm height) filled with opaque water to a 32 cm depth. The tank was conceptually divided into four quadrants, with a platform (30 cm height, 15 cm diameter) in a fixed location. At day 9 after treatment, rats were subjected to four consecutive training trials per day for four consecutive days. The escape latency and the number of crossings over the platform location were counted on days 13 and 15 after paclitaxel treatment. Subsequently, all rats were subjected to a probe trial, where the platform was removed and the number of crossings over the platform location within 60 s was recorded.

### MWT after intrathecal administration

Rats with paclitaxel-induced neuropathic pain were implanted with intrathecal catheters and randomly divided into four groups (*n* = 10/group). Rats in the N group received intrathecal injections of normal saline solution. Rats in the B + N group received intrathecal injections with 10 μL baclofen at the concentration of 0.05 μg/μL (TCI, Tokyo, Japan). Rats in the SN + N and SN + B + N groups received intrathecal injections of SN50 and SN50 plus baclofen, respectively. Rats in the C group received intrathecal injections of saline solution. All rats received three 10 μL injections with a 15 min interval between injections. MWT was determined 120 min before (T2) and after injections (T3).

### Protein expression of GABA_B1_ receptors, NF-κB, IL-1β and TNFα in rats by immunohistochemistry

In each group, five animals were sacrificed at T3, and their whole brain tissues were obtained. The hippocampus was rapidly dissected, fixed, dehydrated, and embedded in paraffin. Sections (5 μm thick) were blocked with 1% H_2_O_2_ and then incubated overnight at 4 °C with primary antibodies, including the anti-GABA_B1_ receptor antibody (1:500, Santa Cruz Technology, Santa Cruz, CA), the anti-NF-κB p65 antibody (1:500, Santa Cruz Technology, Santa Cruz, CA), the anti-IL-1β antibody (1:500, Santa Cruz Technology, Santa Cruz, CA) and the anti-TNFα antibody (1:500, Santa Cruz Technology, Santa Cruz, CA). Next, biotinylated sheep anti-rabbit secondary antibody (Beijing Zhongshan Jinqiao Inc., Beijing, China) was added, and the sections were incubated for 40 min at 37 °C. The reaction product was revealed using the diaminobenzidine developing solution and the true color multi-function CMIAS pathological image-analyzing system (Media Cybernetics, Rockville, MD) was used for image analysis.

### Protein expression of GABA_B1_ receptors, NF-κB, IL-1β and TNFα in the five groups by Western blot

In each group, five animals were sacrificed at T3, and the total protein was extracted from their hippocampal tissues. Protein levels of GABA_B1_ receptors, NF-κB, IL-1β and TNFα were analyzed using western blot assay. Briefly, total protein concentrations were determined using bicinchoninic acid assays (Nanjing Jiancheng Bioengineering Institute, Nanjing, China), and western blot assays were performed as described above. Primary antibodies included the anti-GABA_B1_ receptor antibody (1:500, Santa Cruz Technology, Santa Cruz, CA), the anti-NF-κB p65 antibody (1:500, Santa Cruz Technology, Santa Cruz, CA), the anti-IL-1β antibody (1:500, Santa Cruz Technology, Santa Cruz, CA) and the anti-TNFα antibody (1:500, Santa Cruz Technology, Santa Cruz, CA), as well as the secondary horseradish peroxidase-labeled goat anti-rabbit immunoglobulin (1:4000, PTG Lab, Chicago, IL). The membrane was developed using enhanced chemiluminescence, exposed to X-ray film, and then photographed under UV light using a UVP gel documentation system (UVP, Upland, CA).

### Statistical analysis

All data are expressed as mean ± standard deviation (SD) and were analyzed using SPSS software version 13.0 (SPSS Inc., Chicago, IL). Within-group differences were analyzed using repeated analysis of variance (ANOVA). Between-groups differences were analyzed using one-way ANOVA. A *p* value of <0.05 was considered statistically significant.

## Results

### Paclitaxel concentration-dependent variability in cell viability

The inhibitory effects of paclitaxel on hippocampal neurons were influenced by the reciprocal interaction between exposure time and concentration (*F* = 9.127, *p* < 0.05) (Supplementary Table 1). At a constant concentration of paclitaxel, the cell growth inhibition rate increased over time (*F* = 7.250, *p* < 0.05). At a constant duration of exposure to paclitaxel, the cell growth inhibition rate increased with increasing concentrations of paclitaxel (*F* = 8.068, *p* < 0.05). The IC_50_ value of paclitaxel after a 24-h treatment was 1 µmol/L.

### Cell apoptosis after 24 h of exposure to different concentrations of paclitaxel

**Figure 1.**
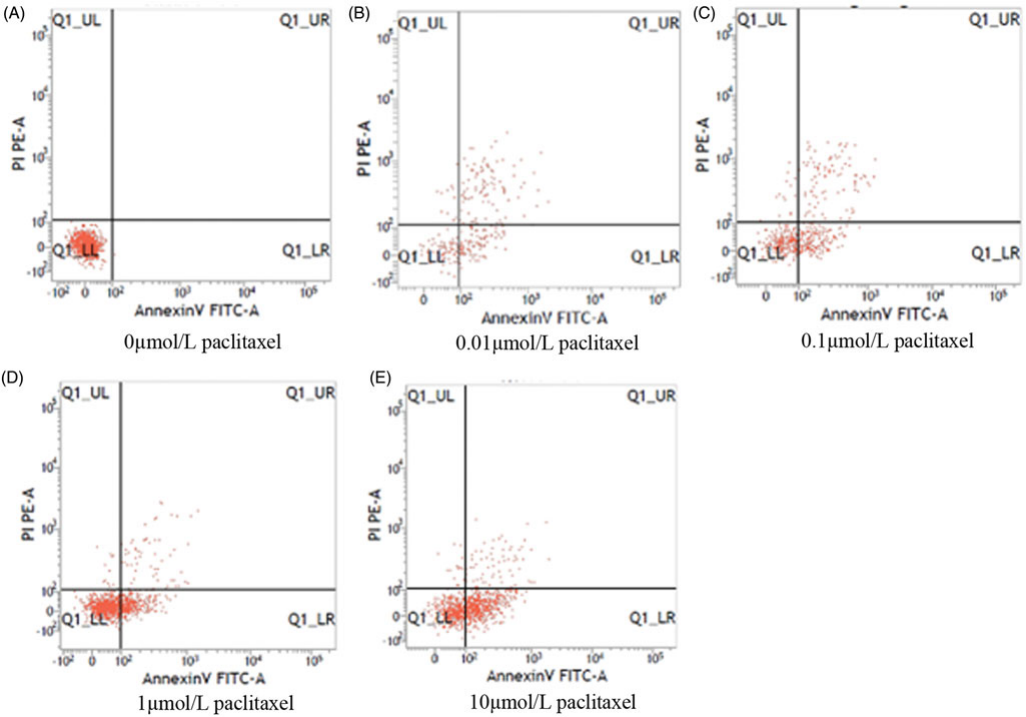
Rate of apoptosis induced by different concentrations of paclitaxel.

To verify the IC_50_ for paclitaxel in hippocampal neurons, a flow cytometry assay was conducted to detect the rate of cell apoptosis induced by different concentrations of paclitaxel (Figure 1 and Table 1). The early apoptotic rate of hippocampal neurons was 48.63 ± 5.76% after 24 h exposure to 1 µmol/L paclitaxel, whereas the apoptotic rate of hippocampal neurons incubated with 10 µmol/L paclitaxel was 68.47 ± 6.75%.

**Figure 3.**
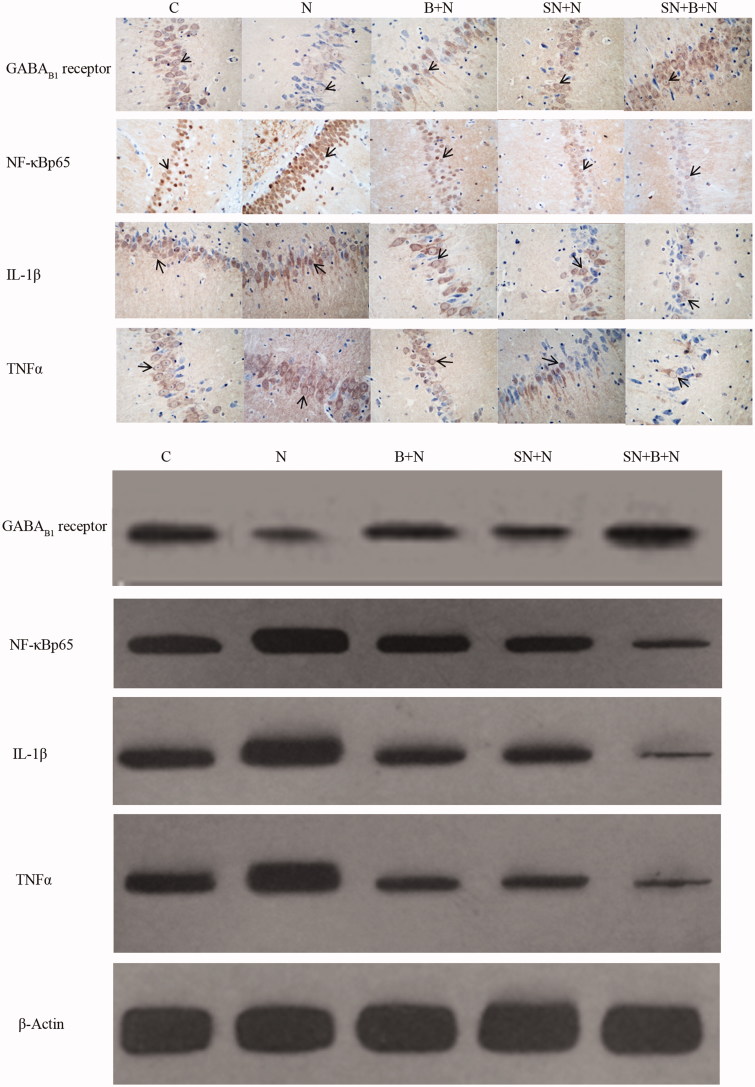
Results of immunohistochemical and western blot assays of NF-κB p65, IL-1β, TNFα and GABA_B1_ receptors in the five groups.

**Table 1.** Cell apoptosis induced by 24 h paclitaxel treatment (%, *n* = 4, ¯x *± s*).

Concentration(µmol/L)	Early apoptosis rate (%)	Late apoptosis rate (%)
0	0	0.29 ± 0.01
0.01	17.90 ± 5.18	9.44 ± 2.37
0.10	38.57 ± 7.39^a^	12.01 ± 2.45^a^
1.00	48.63 ± 5.76^a^	15.88 ± 3.61^a^
10.00	68.47 ± 6.75^a^^b^	18.83 ± 6.23^a^

a *p* < 0.05, compared with paclitaxel of 0.01 µmol/L;

b *p* < 0.05, compared with paclitaxel of 1 µmol/L.

### Morphological changes in hippocampal neurons

The cell bodies of hippocampal neurons, which comprise 1–2 dendrites and a long axon, were cone-, spindle-, or triangular-shaped in the C, SB and SN groups, respectively. In the N group, which was treated with paclitaxel, shrunk cell bodies and shortened axons were observed. Compared with the C group, the numbers of axonal and dendritic branches of neurons were reduced in the N, SB + N and SN + N groups and normal in the SB and SN groups, respectively.

### Protein levels and early apoptosis rate of hippocampal neurons from the six groups

**Figure 2.**
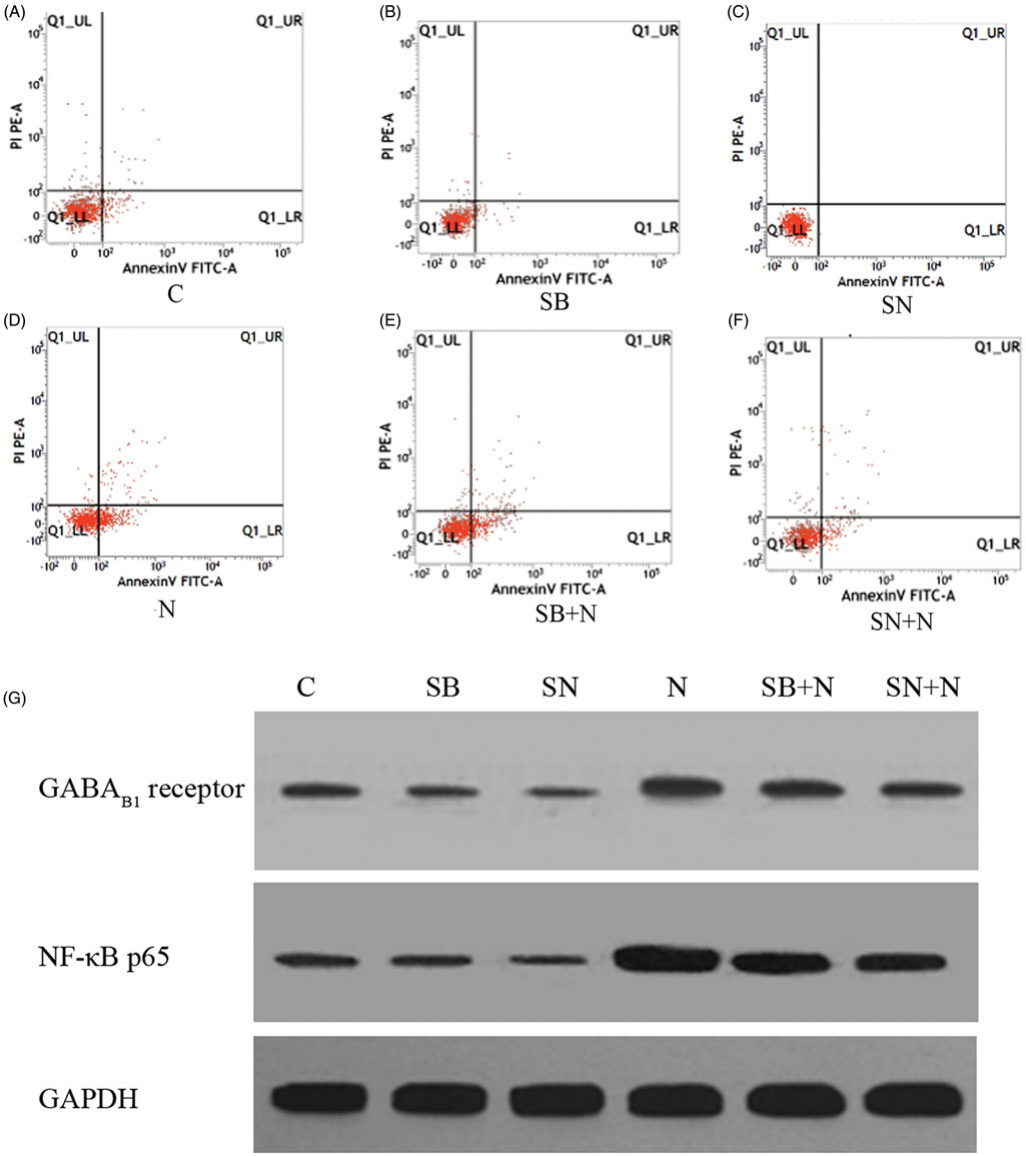
Apoptosis and protein levels of NF-κB p65 and GABA_B1_ receptors in the six groups.

Changes in the early apoptosis rate were consistent with the levels of the expression levels of NF-κB p65 and GABA_B1_ receptor proteins (Figure 2 and Table 2). Compared with the C group, the early apoptosis rate of neurons and the protein levels of NF-κB p65 and GABA_B1_ receptors were increased in the N, SB + N and SN + N groups (all *p* < 0.05) and significantly decreased in the SN group (*p* < 0.05). No significant change was observed in the early apoptosis rate of neurons and the protein level of GABA_B1_ receptors in the SB group (*p* > 0.05). Compared with the N group, the three indicators were significantly decreased (*p* < 0.05) in all five groups, especially in the non-paclitaxel-treated groups (all *p* < 0.05).

**Table 2.** Comparison among early apoptosis rate, and protein levels of GABA_B1_ receptor and NF-κB p65 in six groups (*n* = 5, ¯x *± s*).

Groups	Early apoptosis rate (%)	GABA_B1_ receptor	NF-κB
C	12.57 ± 2.39	0.150 ± 0.032	0.822 ± 0.059
SB	10.36 ± 2.45	0.135 ± 0.023	0.153 ± 0.042^a^
SN	7.91 ± 1.18^a^	0.107 ± 0.026^a^	0.168 ± 0.041^a^
N	49.16 ± 3.12^a,^^b^	0.381 ± 0.014^a,b^	3.452 ± 0.654^a,b^
SB + N	31.18 ± 3.02^a,b,^^c^	0.243 ± 0.013^a,b,^^c^	1.729 ± 0.461^a,b,^^c^
SN + N	28.47 ± 3.75^a,b,^^c^	0.268 ± 0.027^a,b,^^c^	1.604 ± 0.361^a,b,^^c^

a *p* < 0.05, compared with the C group;

b *p* < 0.05, compared with the SB or the SN group;

c *p* < 0.05 compared with the N group.

### Effects of paclitaxel on learning and memory in rat models of paclitaxel-induced neuropathic pain

Compared with normal rat controls, the escape latency and the number of crossings over the platform location in the MWT test were significantly decreased in rat models treated with paclitaxel (*p* < 0.05) (Table 3).

**Table 3.** The escape latency and the number of crossings over the platform location of rats in two groups (*n* = 15 ¯x ± s).

	Escape latency (s)		Number of crossings over the platform location	
Groups	13 d later	15 d later	13 d later	15 d later
C	20.42 ± 2.71	19.51 ± 2.63	6.42 ± 1.13	5.64 ± 1.05
P	29.84 ± 4.12*	24.32 ± 2.94*	3.21 ± 0.82*	3.14 ± 0.63*

*p < 0.05, compared with the C group.

### Changes in MWT values in the rat model at different time points

MWT values at three time points (T1, T2 and T3) were significantly lower in the N, B, SN and SN + B groups than in the C group (*p* < 0.05) (Table 4). In the B, SN and SN + B groups, MWT values at T3 were significantly increased compared with those at T1 and T2 (*p* < 0.05).

**Table 4.** The mechanical withdrawal threshold (MWT) values of rats in different groups at three time points (g, *n* = 10, ¯x ± s).

Groups	T1	T2	T3
C	11.85 ± 1.15	12.02 ± 1.48	11.78 ± 1.04
N	5.73 ± 0.89^a^	5.16 ± 1.14^a^	5.65 ± 0.89^a^
B + N	5.02 ± 0.75^a^	5.56 ± 1.26^a^	9.54 ± 0.78^b,c^
SN + N	5.35 ± 1.04^a^	5.61 ± 1.56^a^	8.11 ± 0.75^b,c^
SN + B+N	5.21 ± 1.18^a^	5.05 ± 1.35^a^	11.15 ± 0.65^b,c,^^d^

a *p* < 0.05, compared with the C group;

b *p* < 0.05, compared with the N group;

c *p* < 0.05, compared with the T1 time point;

d *p* < 0.05, compared with the T2 time point.

### Protein expression levels of GABA_B1_ receptors, NF-κB, IL-1β and TNFα in the five groups

The results of immunohistochemistry assays for proteins are shown in Table 5 and Figure 3. Compared with the C group, the rate of GABA_B1_ receptor-positive cells (8%) was decreased and the rate of NF-κB p65 (27%), IL-1β (27%) and TNFα (32%) positive cells was significantly increased in the N group (*p* < 0.05). Compared with the N group (8%), the rate of GABA_B1_ receptor-positive cells in the B + N (22%), SN + N (20%) and SN + B + N groups (31%) was increased and the rate of NF-κB p65, IL-1β and TNFα positive cells was decreased in the B + N (NF-κB p65: 27% versus 23%, IL-1β: 27% versus 23%, TNFα: 32% versus 25%), SN + N (NF-κB p65: 27% versus 19%, IL-1β: 27% versus 22%, TNFα: 32% versus 22%, and SN + B + N groups (NF-κB p65: 27% versus 13%, IL-1β: 27% versus 13%, TNFα: 32% versus 16%) (*p* < 0.05).

**Table 5.** The number of GABA_B1_ receptor, NF-κB p65, IL-1β and TNFα-positive cells determined by immunohistochemistry assay analysis and western blot results of proteins in five groups (*n* = 5, ¯x ± s).

Groups	GABA_B1_	NF-κB p65	IL-1β	TNFα
Immunohistochemistry assay				
C	11.6 ± 1.5	10.4 ± 1.9	11.4 ± 2.2	11.0 ± 1.6
N	9.0 ± 1.6^a^	29.6 ± 2.4^a^	30.4 ± 3.4^a^	31.0 ± 3.4^a^
B + N	19.4 ± 2.1^b^	21.2 ± 1.5^b^	22.0 ± 1.0^b^	23.0 ± 1.6^b^
SN + N	20.8 ± 1.9^b^	18.6 ± 2.1^b^	19.6 ± 1.8^b^	22.2 ± 0.8^b^
SN + B+N	28.0 ± 1.9^b^	12.6 ± 1.5^b^	14.6 ± 1.5^b^	16.6 ± 1.7^b^
Western blot assay				
C	0.80 ± 0.16	1.10 ± 0.08	1.22 ± 0.10	1.18 ± 0.04
N	0.58 ± 0.12^a^	1.27 ± 0.09^a^	1.52 ± 0.16^a^	1.33 ± 0.07^a^
B + N	0.88 ± 0.03^b^	1.01 ± 0.12^b^	1.07 ± 0.04^b^	0.98 ± 0.07^b^
SN + N	0.79 ± 0.09^b^	0.98 ± 0.10^b^	1.08 ± 0.06^b^	1.00 ± 0.04^b^
SN + B+N	0.95 ± 0.02^b^	0.78 ± 0.17^b^	0.88 ± 0.09^b^	0.71 ± 0.08^b^

a *p* < 0.05, compared with the C group;

b *p* < 0.05, compared with the N group.

The results of western blot assays are shown in Table 5 and Figure 3. Compared with the C group (0.337), protein levels of GABA_B1_ receptor were significantly decreased (N: B + N:SN + N:SN + B + N group =0.216:0.331:0.265:0.379), whereas those of NF-κB p65, IL-1β and TNFα were significantly increased in all other groups (all *p* < 0.05). The protein levels of GABA_B1_ receptor were higher and those of NF-κB p65, IL-1β and TNFα were significantly lower (*p* < 0.05) in the B + N (GABA_B_:0.331, NF-κB p65:0.423, IL-1β:0.406, TNFα:0.397) and SN + N groups (GABA_B_:0.265, NF-κB p65:0.414, IL-1β:0.406 and TNFα:0.387) than in the N group (GABA_B_:0.261, NF-κB p65:0.522, IL-1β:0.550 and TNFα:0.497). In addition, protein levels of GABA_B1_ receptor were higher (B + N:SN + N:SN + B + N group =0.331:0.265:0.379) and those of NF-κB p65 (B + N:SN + N:SN + B + N group =0.423:0.414:0.345), IL-1β (B + N:SN + N: SN + B + N group =0.406:0.406:0.349) and TNFα (B + N: SN + N: SN + B + N group =0.397:0.387:0.274) were lower in all other groups than in the N group (GABA_B_:0.261, NF-κB p65:0.522, IL-1β:0.550, TNFα:0.497) (all *p* < 0.05).

## Discussion

The use of paclitaxel is restricted due to its side effects, particularly neurotoxicity (Frederiks et al. 2015). Therefore, understanding the mechanisms underlying the cytotoxic activity of paclitaxel would be extremely helpful for minimizing or eliminating paclitaxel-induced side effects.

In this study, the exposure time and the optimum concentration of paclitaxel for hippocampal neurons were based on a previous study (Español et al. 2012). Our results revealed a reciprocal relationship between concentration and exposure time, which is consistent with results of previous studies on paclitaxel in cancer cells (Chirio et al. 2014; Bonomi et al. 2015). To validate the reliability of IC_50_ (1 µmol/L), we investigated the apoptotic effects of different concentrations of paclitaxel on hippocampal neurons. Results were largely consistent with results of previous studies (Chirio et al. 2014; Bonomi et al. 2015) and our expectations. Therefore, an IC_50_ of 1 µmol/L for 24 h was used in the next part of our study.

The NF-κB complex contains five subunits (p65, relB, C-rel, NF-κB p51 and NF-κB p52) that form dimeric combinations, of which p65/p60 is the most common heterodimer (Ridder and Schwaninger 2009). The protein levels of GABA_B1_ receptors, which predominantly mediates postsynaptic inhibition (Vigot et al. 2006), and the effects of NF-κB p65 were determined. Our *in vitro* experiments indicated that paclitaxel effectively induced cell apoptosis and increased GABA_B_ receptor expression. This increasing trend was inhibited by SN50 or SB203580, suggesting that the NF-κB pathway and GABA_B_ receptors play important roles in apoptosis. One possible mechanism is that paclitaxel activates the p38MAPK/NF-κB pathway, and proinflammatory NF-κB is activated to promote the release of inflammatory cytokines, including IL-1 and TNFα, leading to neuronal apoptosis (Maqbool et al. 2013). In addition, the binding of TNFα to TRAIL-R1 (DR4) and TRAIL-R2 (DR5) upregulates NF-κB and induces cell apoptosis (Zhu et al. 2014). Moreover, activated GABA_B_ receptors protect against stress-induced apoptosis in neurons by activating the PI3K/AKT pathway (Tu et al. 2010). Another possible mechanism is that elevated NF-κB activity leads to increased production of nitric oxide, which promotes hyperoxide-induced neuronal apoptosis (Cheng et al. 2010). A third possible mechanism is that GABA_B_ receptors participate in the regulation of neuronal apoptosis through the p38MAPK pathway (Jiang et al. 2012) and the NF-κB pathway (Prud'Homme et al. 2013). In this study, GABA_B_ receptor expression was increased by blocking the p38MAPK pathway, which is consistent with the results of the study by Cheng et al. (2010). These results suggest that the p38MAPK/NF-κB pathway plays crucial regulatory roles in GABA_B_ receptor expression during paclitaxel-induced hippocampal neuronal apoptosis.

Additional *in vivo* experiments were conducted to verify the findings described above. Expression of NF-κB and inflammatory cytokines increased in paclitaxel-treated rats. However, the GABA_B_ receptor expression patterns differed from those in *in vitro* experiments. This may be due to different experimental materials; the *in vitro* study used hippocampal neurons from the newborn rats, whereas the *in vivo* study was performed in adult rats. Besides, as described in the previous studies (Luo et al. 2010; Magnaghi and Motta 2012), GABA release may be decreased due to the death of GABAergic neurons under neuropathic pain condition. Although the activation of GABA_B_ receptors can inhibit GABA expression, the apoptosis of GABAergic hippocampal neurons leads to the declined endogenous GABA, which may positively inhibit the GABA_B_ receptor expression. In our paper, rat models of neuropathic pain were more susceptible to baclofen than cells *in vitro*. The effect of baclofen on the increase of GABA_B_ receptors was larger than the decrease of GABA_B_ receptors induced by neuropathic pain. Thus, the level of protein level of GABAB receptors in the B + N group was larger than N group. Besides, the controversial results obtained in this paper should be further validated in a large number of studies. In addition, one limitation of our study is that changes in learning and memory were not investigated *in vivo*, mainly due to time constraints associated with long-term memory formation. Moreover, the swimming ability of rats significantly decreased after the intrathecal administration of paclitaxel, and the results were influenced by this change in the subject rats. The reasons for this will be explored in our next study.

In conclusion, paclitaxel can induce hippocampal neuron apoptosis and neuropathic pain and reduce cognitive function in rats. The p38MAPK/NF-κB pathway, especially downstream components of the NF-κB pathway, may be crucial in regulating the expression of GABA_B_ receptors.

## Supplementary Material

Xiuli_Wang_et_al_supplemental_content.zip
