# Supplementary material for: Role of GABAB receptors and p38MAPK/NF-κB pathway in paclitaxel-induced apoptosis of hippocampal neurons
Source: Pharm Biol. 2017 Nov 8;55(1):2188–95. doi: 10.1080/13880209.2017.1392987 (PMC6130610; doi:10.1080/13880209.2017.1392987)
Supplement: Xiuli_Wang_et_al_supplemental_content.zip [file IPHB_A_1392987_SM5019.zip › Xiuli Wang et al supplemental content.pdf]

项目批准号	81371231
申请代码	H0903
归口管理部门	
依托单位代码	05001708A0398-0757

813712311004797

国家自然科学基金委员会

资助项目计划书

资助类别：面上项目

亚类说明：

附注说明：

项目名称：紫杉醇诱发神经痛中GABAB受体表达及MAPKs通路激活对中枢神经元递质释放的影响

资助经费：70万元　　执行年限：2014.01-2017.12

负 责 人：王秀丽

通讯地址：石家庄市自强路139号

邮政编码：050051　　电　　话：0311-88602072

电子邮件：wangxl301@aliyun.com

依托单位：河北医科大学

联 系 人：吕萍　　电　　话：0311-86266225

填表日期：2013年08月28日

国家自然科学基金委员会制

Version：1.004.797

国家自然科学基金委员会资助项目计划书填报说明

一、项目负责人收到《关于国家自然科学基金资助项目批准及有关事项的通知》（以下简称《批准通知》）后，请认真阅读本填报说明和自然科学基金相关项目及财务管理办法（查阅http://www.nsfc.gov.cn/），按《批准通知》的要求认真填写《国家自然科学基金委员会资助项目计划书》（以下简称《计划书》）。

二、填写《计划书》时要求科学严谨、实事求是、表述清晰、准确。《计划书》经主管科学部审核批准后，将作为项目研究计划执行和检查、验收的依据。

三、《计划书》简表部分自动生成，其他部分按以下要求填写：

（一）各类获资助项目都必须填写中、英文摘要及主题词，按批准经费填报经费预算表。

（二）正文撰写：

1. 对于面上项目、青年科学基金项目、地区科学基金项目，如果《批准通知》中没有修改要求的，只需选择“研究内容和研究目标按照申请书执行”即可；如果《批准通知》中明确要求调整研究内容的，须选择“根据研究方案修改意见更改”并填报相关修改内容。

2. 对于重点项目、重大项目、科学仪器基础研究专款项目及国家重大科研仪器设备研制专项（自由申请）项目，须选择“根据研究方案修改意见更改”，根据《批准通知》的要求填报研究内容，不得自行降低、更改研究目标（或仪器研制指标）或缩减关键的研究内容。此外，还要突出以下几点：
1)研究的难点和在实施过程中可能碰到的问题（或仪器研制风险），拟采用的研究方案和技术路线；
2)项目主要参与者分工，并请说明课题及合作单位之间的关系与分工。

3. 对于国家杰出青年科学基金、优秀青年科学基金和海外及港澳学者合作研究基金项目，须选择“根据研究方案修改意见更改”，按下列提纲撰写：
1）研究方向；
2）
结合国内外研究现状，说明研究工作的学术思想和科学意义（限两个页面）；
3）研究内容、研究方案及预期目标（限两个页面）；
4）分年度进度安排；
5）研究队伍的组成情况。

4. 对于其他类型项目，参照面上项目填报。

简表

<table>
<tr><td rowspan="6">申请者信息</td><td>姓名</td><td>王秀丽</td><td>性别</td><td>女</td><td>出生年月</td><td>1964年09月</td><td>民族</td><td>汉族</td></tr>
<tr><td>学位</td><td colspan="3">博士</td><td>职称</td><td colspan="3">教授</td></tr>
<tr><td>电话</td><td colspan="2">0311-88602072</td><td colspan="2">电子邮件</td><td colspan="3">wangxl301@aliyun.com</td></tr>
<tr><td>传真</td><td colspan="2"></td><td colspan="2">个人网页</td><td colspan="3"></td></tr>
<tr><td>工作单位</td><td colspan="7">河北医科大学</td></tr>
<tr><td>所在院系所</td><td colspan="7">第三临床医院</td></tr>
<tr><td rowspan="3">依托单位信息</td><td>名称</td><td colspan="5">河北医科大学</td><td>代码</td><td>05001103</td></tr>
<tr><td>联系人</td><td colspan="2">吕萍</td><td colspan="2">电子邮件</td><td colspan="3">hbydkjc@126.com</td></tr>
<tr><td>电话</td><td colspan="2">0311-86266225</td><td colspan="2">网站地址</td><td colspan="3">www.hebmu.edu.cn</td></tr>
<tr><td rowspan="3">合作单位信息</td><td colspan="7">单位名称</td><td>代码</td></tr>
<tr><td colspan="7"></td><td></td></tr>
<tr><td colspan="7"></td><td></td></tr>
<tr><td rowspan="7">项目基本信息</td><td>项目名称</td><td colspan="7">紫杉醇诱发神经痛中GABAB受体表达及MAPKs通路激活对中枢神经元递质释放的影响</td></tr>
<tr><td>资助类别</td><td colspan="3">面上项目</td><td colspan="2">亚类说明</td><td colspan="2"></td></tr>
<tr><td>附注说明</td><td colspan="7"></td></tr>
<tr><td>申请代码</td><td colspan="3">H0903</td><td colspan="4">H0907</td></tr>
<tr><td>基地类别</td><td colspan="7"></td></tr>
<tr><td>执行年限</td><td colspan="7">2014.01-2017.12</td></tr>
<tr><td>资助经费</td><td colspan="7">70.0000万元</td></tr>
</table>

项目摘要

中文摘要(500字以内)：
作为抗肿瘤药物，临床紫杉醇诱发神经病理痛很常见，目前尚无有效治疗方法。课题组前期研究表明糖尿病神经痛大鼠脊髓背角神经元GABAB受体表达下调、而p-CREB表达上调，谷氨酸释放增多。目前已证实紫杉醇与糖尿病诱发神经痛有相同病理改变及MAPKs通路激活机制，但紫杉醇诱发神经痛中GABAB受体表达变化及MAPKs通路激活对中枢神经元递质释放的影响尚不清楚。本研究利用选择性GABAB受体激动剂及各种转录因子抑制剂，体外水平研究紫杉醇对GABAB受体、MAPKs通路激活及转录因子表达的影响，阐明NF-kB作为关键靶点对GABAB受体与MAPKs通路调控机制，以及Ca2 +在其中的作用；在此基础上，利用紫杉醇诱发痛大鼠模型和选择性激动剂/抑制剂，探讨中枢GABAB受体表达变化及MAPK通路激活对海马/脊髓背角神经元递质释放的影响。该研究有助于阐明紫杉醇诱发痛的发生机制，为开发针对性药物提供新的思路。

关键词(不超过5个，用分号分开)： 疼痛 ;GABA-B受体 ;MAPKs通路 ;神经病变 ;紫杉醇

Abstract(limited to 500 words)：
It is quite common that paclitaxel, an antitumor agent, can induce neuropathic pain, but there are no effective treatments in clinical practice. The previous studies showed that there are down-regulation of GABAB receptors, up-regulation of p-CREB and increased glutamic acid neurotransmitter release in spinal cord dorsal horn neurons in rats with diabeteic neuropathic pain. It has been established that there are the same pathological changes and activation mechanism of MAPK pathway in paclitaxel and diabeteic induced neuropathic pain, but in paclitaxel-induced neuropathic pain, the effect of GABAB receptors expression and MAPKs pathway activatation on central neurotransmitter release is not clear. To explore the issue, by using selective GABAB receptors agonist and transcription factors antagonists, the study is to investigate the effect of paclitaxel on expression of GABAB receptors, activation of MAPKs pathway and expression of transcription factor in vitro, further to elucidate that NF-kB is the key target of mutual regulation of GABAB receptors and MAPKs pathway , and to explore the role of Ca2 + in this progress. Based on vitro experiments, by using model rats with paclitaxel-induced neuropatic pain and selective agonist/antagonist , this study is to explore the role of central GABAB receptors expression and MAPKs pathway activatation in neurotransmitter release in neurons of hippocampal and spinal cord dorsal horn in rats with paclitaxel-induced pain. This study is desiged to elucidate the pathogenesis in paclitaxel-induced neuropathic pain, and to provide new ideas for the further development of targeted medicines.

Keywords(limited to 5 keywords,seperated by;)： pain ;GABA-B receptors ;MAPKs pathway ;neuropathy ;paclitaxel

项目组主要成员

编号	姓名	出生年月	性别	职称	学位	单位名称	电话	电子邮件	项目分工	每年工作时间（月）
1	王秀丽	1964.09	女	教授	博士	河北医科大学	0311-88602072	wangxl301@aliyun.com	项目负责人	8
2	郭跃先	1964.9	男	教授	硕士	河北医科大学	0311-88602126	guoyuexian@yahoo.com.cn	神经电生理、行为学	7
3	李德培	1969.11	男	副教授	硕士	The University of Texes	0311-88602172	dpli@yahoo.com.cn	电生理、分子生物学	6
4	刘飞飞	1984.10	男	医师	硕士	河北医科大学	0311-88602072	feiliulove@soho.com	分子生物学、行为学	7
5	任伟	1986.11	女	硕士生	学士	河北医科大学	0311-88602072	renwei@yahoo.com.cn	分子生物学、电生理	10
6	白慧萍	1987.5	女	硕士生	学士	河北医科大学	0311-88602072	baihuiping@yahoo.com.cn	分子生物学、电生理	8
7	金子	1988.6	女	硕士生	学士	河北医科大学	0311-88602072	jinzi@yahoo.com.cn	行为学、分子生物学	10
8	李志华	1976.6	女	主治医师	博士	河北医科大学	0311-88602072	lizhihua@yahoo.com.cn	分子生物学、电生理	8
9	刘朋	1980.2	男	硕士生	学士	河北医科大学	0311-88602072	liupeng@yahoo.com.cn	电生理、分子生物学	8
10	郝立净	1980.5	女	博士生	硕士	河北医科大学	0311-88602072	haolijing@yahoo.com.cn	行为学、分子生物学	10

总人数	高级	中级	初级	博士后	博士生	硕士生
10	3	1	1		1	4

经费预算表

（金额单位:万元）

预算编制说明：

1. 在填报本表之前，请根据项目资助类别认真阅读相关的资助经费管理办法；经费预算的编制以申请书中 的《经费申请表》为基础，以《国家自然科学基金项目资助批准通知书》中的资助金额为依据；
2. 编制经费预算时，不考虑不可预见因素和前期投入；
3. 购置与试制仪器设备在5万元以上（包括5万元）时，须在报告正文中逐项说明用途和必要性。

科目	预算经费	备注（计算依据与说明）
一. 研究经费	52.1000	
1. 科研业务费	6.5000	
(1) 测试/计算/分析费	1.5000	统计分析、仪器测试调试、图像资料分析
(2) 能源/动力费	1.5000	实验室日常用水、电消耗
(3) 会议费/差旅费	2.0000	参加国内外学术会议、成果申报
(4)出版物/文献/信息传播事务费	1.5000	中英文论文版面费、文献检索、查新等
(5) 其他	0	
2. 实验室材料费	40.0000	
(1) 原材料/试剂/药品购置费	38.0000	实验动物、试剂、各种受体激动剂/抑制剂
(2) 其他	2.0000	实验过程中的试剂药品材料消耗
3. 仪器设备费	4.1000	
(1) 购置	2.1000	膜片钳记录系统的各种耗材如：电极、导线、
(2) 试制	2.0000	实验室设备的维修保养
4. 实验室改装费	1.5000	对电生理操作平台的维修改造
5. 协作费	0	
二. 国际合作与交流费	6.0000	
1. 出境国际旅费	3.0000	项目组成员出国技术交流
2. 境外合作人员来华生活费	3.0000	接待境外专家来访
3. 来华举办学术会议费	0	
4. 其他	0	
三. 劳务费	8.4000	直接参加项目研究的研究生、博士后人员的劳务费用
四. 管理费	3.5000	不得超过预算经费的5%
合 计	70.0000	

与本项目相关的其他经费来源	国家其他计划资助经费	
	其他经费资助(含部门匹配)	
	其他经费来源合计	

报告正文

研究内容和研究目标按照申请书执行。

国家自然科学基金资助项目签批审核表

我接受国家自然科学基金的资助，将按照申请书、项目批准意见和计划书负责实施本项目（批准号：81371231），严格遵守国家自然科学基金委员会关于资助项目管理、财务等各项规定，切实保证研究工作时间，认真开展研究工作，按时报送有关材料，及时报告重大情况变动，对资助项目发表的论著和取得的研究成果按规定进行标注。

项目负责人（签章）：

年　月　日

我单位同意承担上述国家自然科学基金项目，将保证项目负责人及其研究队伍的稳定和研究项目实施所需的条件，严格遵守国家自然科学基金委员会有关资助项目管理、财务等各项规定，并督促实施。

依托单位（公章）

年　月　日

本栏目由基金委填写

科学处审查意见：

建议年度拨款计划（本栏目为自动生成，单位：万元）：

年度	总额	第一年	第二年	第三年	第四年	第五年
金额						

负责人（签章）：

年　月　日

科学部审查意见：

负责人（签章）：

年　月　日

本栏目主要用于重大项目等

相关局室审核意见：

负责人（签章）：

年　月　日

委领导审批意见：

委领导（签章）：

年　月　日

关于国家自然科学基金资助项目批准及有关事项的通知

河北医科大学 王秀丽先生/女士:

根据《国家自然科学基金条例》的规定和专家评审意见，国家自然科学基金委员会（以下简称自然科学基金委）决定批准资助您的申请项目。项目批准号: 81371231 ，项目名称 紫杉醇诱发神经痛中 GABAB 受体表达及 MAPKs 通路激活对中枢神经元递质释放的影响 ,资助金额 70.00 万元,项目起止年月: 2014 年 01 月至 2017 年 12 月，有关项目的评审意见及修改意见附后。

请尽早登录科学基金网络信息系统（https://isis.nsfc.gov.cn），获取《国家自然科学基金资助项目研究计划书》（以下简称计划书）并按要求填写。计划书电子文件通过科学基金网络信息系统（https://isis.nsfc.gov.cn）上传，由依托单位确认后，自然科学基金委进行审核；计划书纸质文件（一式两份）由依托单位审核并加盖单位公章后报送至自然科学基金委项目材料接收工作组。

自然科学基金委接收依托单位提交计划书电子版截止时间为 **2013 年 9 月 11 日 16 点前**，提交计划书电子修改版截止时间为 **2013 年 9 月 18 日 16 点前**；计划书纸质版于计划书电子版通过自然科学基金委审核后再行打印（建议双面打印），自然科学基金委接收计划书纸质版截止时间为 **2013 年 9 月 27 日 16 点前**。

请按照依托单位规定时间，及时将计划书电子版和纸质版先后提交依托单位进行确认审核。对于有修改意见的项目，请按修改意见及时调整计划书相关内容；如对修改意见有异议，须在计划书电子版报送截止日期前提出。计划书电子文件和纸质文件内容应当保证一致。

未说明理由且逾期不报计划书者，视为自动放弃接受资助。

附件：项目评审意见及修改意见

国家自然科学基金委员会

医学科学部

2013 年 08 月 15 日

附件：项目评审意见及修改意见表

项目批准号	81371231	项目负责人	王秀丽	申请代码 1	H0903
项目名称	紫杉醇诱发神经痛中 GABAB 受体表达及 MAPKs 通路激活对中枢神经元递质释放的影响				
资助类别	面上项目		亚类说明	常规面上项目	
附注说明					
依托单位	河北医科大学				
资助金额	70.00 万元		起止年月	2014 年 01 月至 2017 年 12 月	

通讯评审意见：

<1>该项目研究紫杉醇诱发神经痛中 GABAB 受体表达以及对 MAPKs 通路的激活机制。研究目标明确，实验方案设计合理。课题组负责人前期在国家自然基金的支持下做出了一定的研究成绩，希望在今后的研究工作中再接再励，研究水平更上一层楼。

<2>申请人已完成一项国家自然基金面上项目，研究脊髓背角 GABAb 受体对糖尿病神经病理痛的调控作用。本次申请的项目拟研究 GABAb 受体和 MAPK 通路对紫杉醇诱发的神经病理痛的调节机制。课题的立题尚有新意，实验设计和技术路线可行，申请人对脊髓背角的 GABAb 受体对疼痛调控作用的研究手段比较熟悉。

<3>1 该项目以紫杉醇诱发神经性痛大鼠模型上研究 GABA-B 受体的表达变化及 MAPK 信号通路在其中的作用,项目立题较新颖，具有一定的理论意义。

2 该项目的研究标比较明确，总体研究方案基本可行, 并有较多的相关前期研究工作结果.

3 申请人具有较好的研究工作基础, 并有较多的相关研究论文发表.

对研究方案的修改意见：

医学科学部

2013 年 08 月 15 日
